# Validation of *GDAP1* and *HECW2* as Epigenetic Markers of Alcohol Use Disorder in Blood and Brain

**DOI:** 10.3390/ijms262210840

**Published:** 2025-11-08

**Authors:** Ariane Wiegand, Marion Friske, Susanne Edelmann, Annika Bender, Lea Fischer, Peter Zill, Gabriele Koller, Georgy Bakalkin, Wolfgang H. Sommer, Anita C. Hansson, Vanessa Nieratschker

**Affiliations:** 1 Max Planck Institute of Psychiatry, 80804 Munich, Germany; 2 Department of Psychiatry and Psychotherapy, Tuebingen Center for Mental Health (TüCMH), University Hospital of Tuebingen, Eberhard Karls University of Tuebingen, 72076 Tuebingen, Germany; 3 German Center for Mental Health, Partner Site Munich-Augsburg, Germany; 4 Institute of Psychopharmacology, Central Institute of Mental Health, Medical Faculty Mannheim, Heidelberg University, 68159 Mannheim, Germany; 5 Waggoner Center for Alcohol and Addiction Research, University of Texas at Austin, Austin, TX 78712, USA; 6 German Center for Mental Health (DZPG), Partner Site Tuebingen, Germany; 7 Graduate School of Cellular and Molecular Neuroscience, Eberhard Karls University of Tuebingen, 72076 Tuebingen, Germany; 8 Institute of Human Genetics, University Hospital Bonn, University of Bonn, 53127 Bonn, Germany; 9 Department of Psychiatry and Psychotherapy, University Hospital, Ludwig Maximilian University, 80336 Munich, Germany; 10 Department of Pharmaceutical Biosciences, Uppsala University, SE-752 37 Uppsala, Sweden; 11 Bethania Hospital for Psychiatry, Psychosomatics and Psychotherapy, 17489 Greifswald, Germany; 12 German Center for Mental Health (DZPG), Partner Site Mannheim-Heidelberg-Ulm, Germany

**Keywords:** DNA methylation, alcohol use disorder (AUD), whole blood, postmortem brain, AUD animal model, *HECW2*, *GDAP1*, epigenetics

## Abstract

Alcohol use disorder (AUD) is associated with widespread epigenetic alterations, including changes in DNA methylation (DNAm). This multi-cohort study validated and extended previous findings on DNAm of *HECW2* and *GDAP1* in AUD, assessed sex differences, and explored DNAm in blood and brain tissue in humans and rats. DNAm was measured via pyrosequencing in human blood (N_Ctrl_ = 341, N_AUD_ = 258), postmortem frontal cortex (Brodmann area 9; discovery cohort: N_Ctrl_ = 10, N_AUD_ = 13, replication cohort: N_Ctrl_ = 64, N_AUD_ = 55) and rat blood and medial prefrontal cortex (N_Ctrl_ = 16, N_AUD_ = 15). Gene expression was assessed in human postmortem brain by quantitative real-time PCR. AUD-associated DNAm differences in *HECW2* and *GDAP1* were replicated in human blood. While decreased *GDAP1* DNAm was only observed in men, *HECW2* hypomethylation was present in both sexes. In brain tissue, initial DNAm increases in AUD and *HECW2* gene expression decreases were not validated in the replication cohort. In rats, *HECW2* hypomethylation appeared in the prelimbic cortex but not in blood. Our findings support the involvement of *HECW2* and *GDAP1* DNAm in AUD, revealing sex-specific and tissue-dependent epigenetic patterns. The opposing DNAm directionality in blood and brain underscores the complexity of alcohol-related epigenetic modifications and suggests the need for multi-tissue, cross-species, and longitudinal studies to clarify causal mechanisms.

## 1. Introduction

Alcohol use disorder (AUD) is a severe mental health condition characterized by mental and physical health problems. Harmful use of alcohol was related to 4.7% of all deaths worldwide and accounted for 4.6% of all disability-adjusted life years (DALYs) in 2019 [1]. Men are 1.5 to 2 times more likely to be affected than women, although this gap has been narrowing over the past few decades [2].

The complex pathogenesis of AUD involves environmental and genetic factors, with heritability estimated at approximately 50% [3], but identified genetic variants account for only a small portion. Notably, sex differences have been observed in the heritability and genetic architecture of AUD, suggesting that males and females may differ in their vulnerability and in the underlying biological pathways [2]. These disparities likely reflect sex-specific gene regulation and neurobiological mechanisms; for example, male and female individuals differ in dopaminergic reward circuitry and GABAergic signaling [4]. Furthermore, sex differences are evident in the clinical manifestations, comorbidity patterns, and treatment responses associated with AUD, highlighting the importance of considering sex as a biological variable in etiological studies [2].

Epigenetic mechanisms, especially DNA methylation (DNAm), may help explain gene–environment interactions in AUD [5,6]. DNAm occurs when a methyl group is added to DNA without altering its sequence, typically at CpG sites [7]. Besides genetic variants and environmental factors which may influence DNAm patterns, alcohol itself also has been shown to impact DNAm. Over the past years, several studies have revealed changes in DNAm in response to acute and chronic alcohol consumption [8,9]. Thus, DNAm may play a role in both the development and manifestation of AUD.

A comprehensive epigenome-wide association study (EWAS) has revealed hundreds of CpG sites that displayed differential DNAm in human blood associated with current heavy alcohol intake [10]. More recently, another extensive EWAS discovered over 2500 differentially methylated CpG sites specifically linked to alcohol consumption [11]. Additionally, further EWAS with smaller sample sizes identified CpG sites associated with AUD both in peripheral tissue such as in saliva [12] and blood cells [13,14,15] as well as in postmortem brain samples [16,17,18,19,20]. However, many findings have not been replicable [21]. To identify robust DNAm patterns, it is important to validate findings in independent cohorts.

In this study, we aim to replicate findings in two genes, *GDAP1* (encoding Ganglioside-induced Differentiation Associated Protein 1) and *HECW2*, (encoding HECT, C2 and WW domain containing E3 ubiquitin protein ligase 2) which have repeatedly been identified to be differentially methylated in multiple cohorts in AUD patients compared to healthy controls [14,15,18,22,23,24]. *GDAP1* and *HECW2* are critical genes involved in neuronal function and development, with *GDAP1* playing a key role in mitochondrial dynamics and neurodegeneration [25], while *HECW2* regulates protein degradation, cell division, and neural signaling, and is associated with neurodevelopmental disorders and cancer [26]. We first replicated previous findings in a larger independent human blood cohort. Next, we assessed whether these patterns could also be observed in human postmortem brain tissue, given its central role in AUD. Moreover, we investigated whether potential DNAm differences in the human postmortem brain were also reflected in differences in gene expression. Due to the inaccessibility of living brain tissue in humans, it is impossible to investigate blood and brain tissue from the same individuals in order to establish a correlation between epigenetic findings in blood and brain. Therefore, lastly, we analyzed the DNAm of these two genes in blood and brain samples derived from a rat model of AUD.

## 2. Results

### 2.1. DNA methylation (DNAm) in Human Whole Blood

To characterize the study sample and examine potential group differences, demographic variables were first compared between AUD patients and controls. There was no significant age difference between the groups (*t*(592.17) = −1.15, *p* = 0.25). However, fewer females were present in the AUD group (χ^2^ = 12.59, *p* < 0.001).

**Figure 1:**
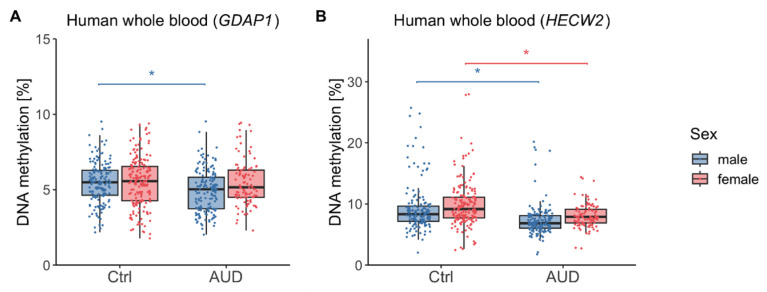
Human whole blood DNA methylation in AUD patients and control participants. DNA methylation distributions in male and female participants within the *GDAP1* gene promoter region (**A**) and *HECW2* intragenic region (**B**). Asterisks indicate statistically significant differences. Ctrl: controls, AUD: alcohol use disorder.

We next assessed group differences in DNAm levels and found that AUD patients showed reduced *GDAP1* DNAm compared to control participants (*F*(1,591) = 8.95, *p* = 0.003, η^2^ = 0.01). Moreover, there was a significant effect of sex driven by higher *GDAP1* DNAm in female as compared to male participants (*F*(1,591) = 3.97, *p* = 0.047, η^2^ = 0.01) and an interaction between AUD and sex (*F*(1,591) = 4.40, *p* = 0.036, η^2^ = 0.01). Post hoc *t*-tests showed that reduced DNAm was only present in male (*t*(313.6) = 3.55, *p* < 0.001, Cohen’s d = 0.40, mean ± SD DNAm_Ctrl,male_ = 5.48% ± 1.35%, mean ± SD DNAm_AUD,male_ = 4.93% ± 1.40%) but not in female participants (*t*(219.1) = 0.13, *p* = 0.90, mean ± SD DNAm_Ctrl,female_ = 5.50% ± 1.65%, mean ± SD DNAm_AUD,female_ = 5.47% ± 1.52%) (Figure 1A). Moreover, *GDAP1* DNAm was significantly associated with chronological age (*F*(1,591) = 4.25, *p* = 0.040, η^2^ = 0.01), with higher levels in older participants (r = 0.09, *p* = 0.024).

For *HECW2*, there was a significant effect of AUD (*F*(1,584) = 58.14, *p* < 0.001, η^2^ = 0.09) and sex (*F*(1,584) = 8.83, *p* = 0.003, η^2^ = 0.01). There was no interaction between these two factors (*F*(1,584) = 0.07, *p* = 0.79). *HECW2* DNAm was decreased in both male (*t*(271.0) = 5.20, *p* < 0.001, Cohen’s d = 0.59, mean ± SD DNAm_AUD,male_ = 7.33% ± 2.59%) and female (*t*(271.8) = 5.36, *p* < 0.001, Cohen’s d = 0.57, mean ± SD DNAm_AUD,female_ = 8.07% ± 1.95%) AUD patients compared to control participants (mean ± SD DNAm_Ctrl,male_ = 9.29% ± 3.95%, mean ± SD DNAm_Ctrl,female =_ 9.90% ± 3.72%) (Figure 1B). Moreover, *HECW2* DNAm was significantly associated with chronological age (*F*(1,584) = 8.67, *p* = 0.003, η^2^ = 0.01), with lower methylation levels observed in older participants (r = −0.16, *p* < 0.001).

There was a linear effect of patients’ daily drinking quantity on *HECW2* (r = −0.17, *p* = 0.007) but not *GDAP1* DNAm (r = −0.06, *p* = 0.37). Moreover, there was no difference in psychotropic medication in patients, neither in *GDAP1* (*t*(236) = −0.60, *p* = 0.55) nor *HECW2* DNAm (*t*(233) = −0.02, *p* = 0.98).

Lastly, DNAm was not correlated with daily cigarette consumption in *GDAP1* (r = −0.08, *p* = 0.23) or *HECW2* (r = −0.09, *p* = 0.18), nor with body mass index (BMI) in *GDAP1* (r = 0.08, *p* = 0.19) or *HECW2* (r = 0.03, *p* = 0.68).

### 2.2. DNAm in Human Postmortem Brain

To complement the findings from whole blood, DNAm was next examined in human postmortem brain tissue (Brodmann area 9). There were no differences in age, blood alcohol at time of death, brain pH, postmortem interval (PMI) and smoking between the discovery and replication cohorts (Supplementary Table S1). Sex differences were inherent, since the discovery cohort included only male individuals.

Participants with AUD and control individuals did not differ significantly in age, sex, brain pH and PMI, neither in the discovery nor the replication cohort. However, there were significant differences between AUD patients and control individuals in smoking in both cohorts and blood alcohol at time of death in the replication cohort (Supplementary Table S1).

**Figure 2:**
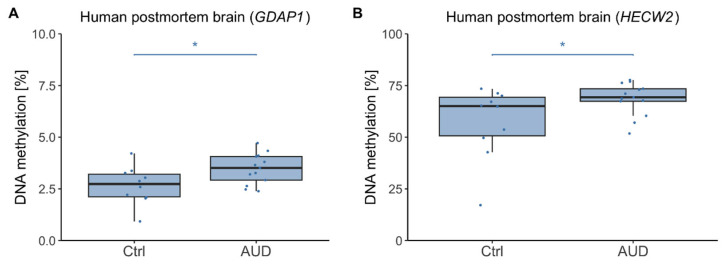
Human postmortem brain DNA methylation in AUD patients and control participants (discovery cohort). DNA methylation distributions in AUD patients and control individuals from the discovery cohort (male only) within the *GDAP1* gene promoter region (**A**) and *HECW2* intragenic region (**B**). Asterisks indicate statistically significant differences. Ctrl: controls, AUD: alcohol use disorder.

We next analyzed DNAm levels in human postmortem brain tissue. In the discovery cohort, *GDAP1* DNAm was significantly increased in AUD patients as compared to control individuals (*F*(1,20) = 8.37, *p* = 0.009, η^2^ = 0.30, mean ± SD DNAm_AUD_ = 3.47% ± 0.74%, mean ± SD DNAm_Ctrl_ = 2.66% ± 0.91%) (Figure 2A). Moreover, there was a significant association with age (*F*(1,20) = 11.69, *p* = 0.003, η^2^ = 0.37) driven by higher *GDAP1* DNAm with increasing age (r = 0.45, *p* = 0.032). Similarly, for *HECW2*, DNAm was significantly increased in AUD patients (*F*(1,20) = 5.03, *p* = 0.036, η^2^ = 0.20, mean ± SD DNAm_AUD_ = 68.53% ± 7.89%, mean ± SD DNAm_Ctrl_ = 57.53% ± 17.44%) (Figure 2B) and there was a significant association with age (*F*(1,20) = 5.62, *p* = 0.028, η^2^ = 0.22), although the correlation of *HECW2* DNAm and age was not significant (r = 0.35, *p* = 0.11).

**Figure 3:**
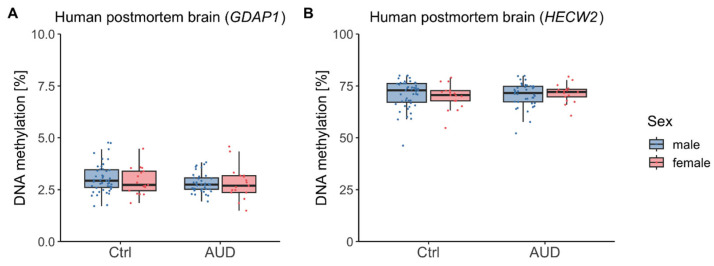
Human postmortem brain DNA methylation in AUD patients and control participants (replication cohort). DNA methylation distributions in male and female participants within the *GDAP1* gene promoter region (**A**) and *HECW2* intragenic region (**B**). Ctrl: controls, AUD: alcohol use disorder.

In the replication cohort, *GDAP1* DNAm showed no significant differences by AUD (*F*(1,114) = 3.06, *p* = 0.08), sex (*F*(1,114) = 0.22, *p* = 0.64), or their interaction (*F*(1,114) = 0.29, *p* = 0.59) (Figure 3A). However, there was a significant association with age (*F*(1,114) = 13.62, *p* < 0.001, η^2^ = 0.11) driven by higher *GDAP1* DNAm with increasing age (r = 0.32, *p* < 0.001). Similarly, for *HECW2* DNAm, there was neither a significant difference with regard to AUD (*F*(1,114) = 0.07, *p* = 0.80), nor an effect of sex (*F*(1,114) = 0.02, *p* = 0.89), nor an interaction between AUD and sex (*F*(1,114) = 0.64, *p* = 0.42) (Figure 3B).

No significant association between DNAm and potentially confounding factors such as PMI, brain pH, blood alcohol at time of death and smoking behavior was found in either the discovery cohort or the replication cohort (see Supplementary Materials).

### 2.3. Gene Expression in Human Postmortem Brain

**Figure 4:**
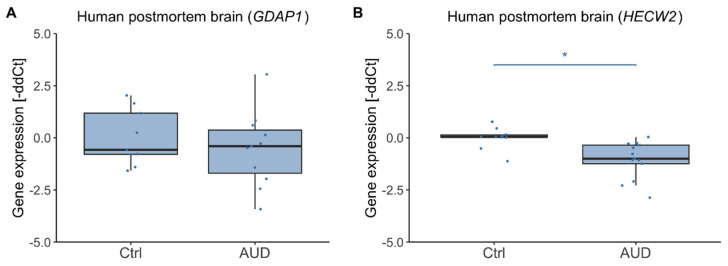
Human postmortem brain gene expression in AUD patients and control participants (discovery cohort). Gene expression distributions in AUD patients and control individuals from the discovery cohort (male only) within the *GDAP1* (**A**) and *HECW2* gene (**B**). Asterisks indicate statistically significant differences. Ctrl: controls, AUD: alcohol use disorder.

To explore whether the observed alterations in DNA methylation were accompanied by transcriptional changes, gene expression was next analyzed in human postmortem brain tissue. In the discovery cohort, *HECW2* transcription was reduced in participants with AUD (*F*(1,20) = 15.43, *p* < 0.001, η^2^ = 0.44, mean ± SD gene expression_AUD_ = −1.05% ± 0.88%, mean ± SD gene expression_Ctrl_ = 0.00% ± 0.51%) while *GDAP1* showed no difference (*F*(1,17) = 0.56, *p* = 0.46, mean ± SD gene expression_AUD_ = 0.53% ± 1.77%, mean ± SD gene expression_Ctrl_ = 0.00% ± 1.34%) (Figure 4). Gene expression was not significantly associated with age for *GDAP1* (*F*(1,17) = 1.70, *p* = 0.21) but was for *HECW2* (*F*(1,20) = 8.58, *p* = 0.008, η^2^=0.30), though the correlation between *HECW2* expression and age was not significant (r = −0.33, *p* = 0.13).

**Figure 5:**
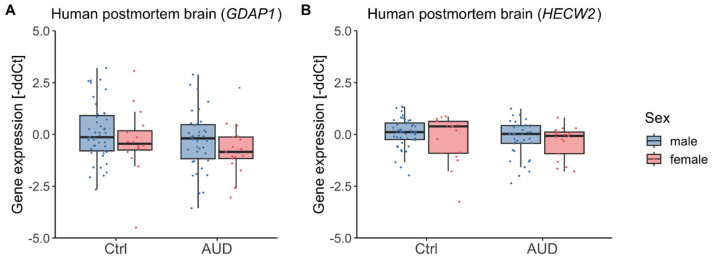
Human postmortem brain gene expression in AUD patients and control participants (replication cohort). Gene expression distributions in male and female participants within the *GDAP1* (**A**) and *HECW2* gene (**B**). Ctrl: controls, AUD: alcohol use disorder.

In the replication cohort, there was no effect of AUD on gene expression, neither for *GDAP1* (*F*(1,113) = 2.70, *p* = 0.10) nor for *HECW2* (*F*(1,114) = 2.52, *p* = 0.12) (Figure 5). Moreover, there were no significant effects of sex (*GDAP1*: *F*(1,113) = 2.35, *p* = 0.13; *HECW2*: *F*(1,114) = 1.97, *p* = 0.16), age (*GDAP1*: *F*(1,113) = 1.80, *p* = 0.18; *HECW2*: *F*(1,114) = 0.55, *p* = 0.46), nor an interaction of sex and AUD (*GDAP1*: *F*(1,113) = 0.01, *p* = 0.94; *HECW2*: *F*(1,114) = 0.12, *p* = 0.73).

Any associations of gene expression with potentially confounding factors are reported in the Supplementary Materials.

### 2.4. DNAm in Whole Blood and Brain Samples from an Alcohol Use Disorder (AUD) Animal Model

**Figure 6:**
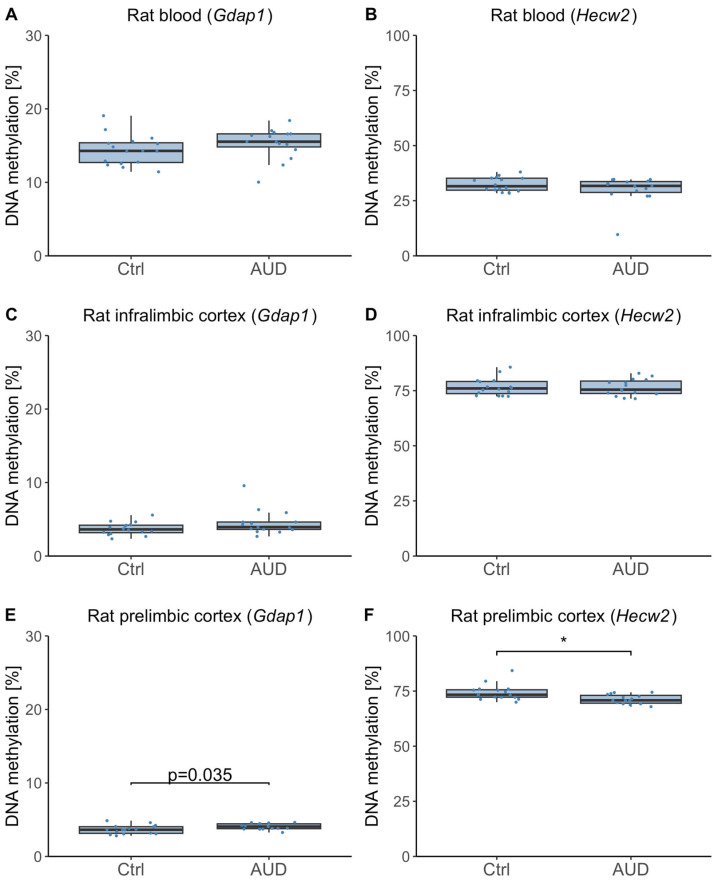
Rat blood and brain DNA methylation. DNA methylation distributions in control and dependent rats within the *Gdap1* gene promoter region (**A**,**C**,**E**) and *Hecw2* intragenic region (**B**,**D**,**F**). Asterisks indicate statistically significant differences after Bonferroni correction for multiple testing. Ctrl: controls, AUD: alcohol use disorder.

Because we observed differing DNAm effects in human whole blood and postmortem brain tissue, we next examined DNAm in an AUD rat model, which allowed paired analysis of blood and brain samples from the same individuals. No significant DNAm differences were observed in whole blood of rats, neither for *Gdap1* (*F*(1,29) = −1.57, *p* = 0.22, Figure 6A) nor for *Hecw2* (*F*(1,29) = 1.69, *p* = 0.20, Figure 6B). Moreover, there were no significant DNAm differences in the infralimbic cortex, neither for *Gdap1* (*F*(1,29) = 2.82, *p* = 0.10, Figure 6C) nor for *Hecw2* (*F*(1,29) = 0.02, *p* = 0.88, Figure 6D). In the prelimbic cortex, there was a nominally significant difference in *Gdap1* DNAm (*F*(1,29) = 4.92, *p* = 0.035, mean ± SD DNAm_AUD_ = 4.07% ± 0.41%, mean ± SD DNAm_Ctrl_ = 3.66% ± 0.59%, Figure 6E). However, this difference did not survive correction for multiple testing. Furthermore, *Hecw2* DNAm in the prelimbic cortex was significantly reduced in dependent rats (*F*(1,29) = 8.47, *p* = 0.007, η^2^ = 0.44, mean ± SD DNAm_AUD_ = 71.31% ± 2.11%, mean ± SD DNAm_Ctrl_ = 74.39% ± 3.54%, Figure 6F).

## 3. Discussion

This study validates and expands on previous evidence linking DNAm of *HECW2* and *GDAP1* to AUD. In a large, independent whole blood cohort, we replicated earlier findings of differential DNAm, previously investigated and observed only in men [23]. Expanding the analysis to include sex differences, we confirmed DNAm associations with AUD in men for both genes but found comparable effects in women only for *HECW2*. To assess the relevance of these changes to AUD neuropathology, we examined DNAm in postmortem brain tissue. While a discovery cohort revealed differential DNAm for both genes, these effects were not replicated in a larger cohort. Lastly, using a rat model of AUD to analyze DNAm in both blood and brain tissue from the same animals, we identified differential methylation only in the prelimbic cortex. Collectively, these findings reinforce the potential involvement of *GDAP1* and *HECW2* DNAm in AUD pathophysiology, while highlighting its complex, tissue-specific, and sex-dependent nature.

### 3.1. DNAm Findings in Human Whole Blood

Replication of previous findings in an independent whole blood cohort strengthens evidence for the association of *HECW2* and *GDAP1* DNAm with AUD. This study expanded on prior research by analyzing a larger cohort including both male and female participants, addressing the limitation of limited sample sizes and male-only cohorts in earlier work. Consistent with earlier research, we observed *GDAP1* hypomethylation in AUD [14,23,24], with post hoc analyses revealing this effect was exclusive to men. This aligns with Dugué et al. (2021), who identified reduced DNAm at the same CpG site (cg23779890) in relation to recent alcohol intake in a large EWAS, although they did not report sex-specific effects [27]. Moreover, recent findings of *GDAP1* hypomethylation in AUD patients’ saliva further support the presence of this epigenetic alteration across multiple tissues [24]. Additionally, *GDAP1* has been proposed as a more general biomarker for substance addiction, since its expression was significantly affected by nicotine exposure in the mouse hippocampus [28]. However, our analyses ruled out smoking as a confounding factor, since no relationship was observed between smoking behavior and *GDAP1* DNAm. Furthermore, the smoking-matched design of our prior study reinforced the specificity of *GDAP1* DNAm changes in AUD.

For *HECW2*, DNAm was reduced in both men and women, consistent with previous findings [14]. This association is further corroborated by Witt et al. (2020), who also reported decreased DNAm in AUD patients, albeit at different sites within *HECW2* [15]. Moreover, Lohoff et al. (2021) identified the same CpG site (cg18752527) as one of the most robust AUD-associated loci across multiple large cohorts [18], noting stronger effects in females, suggesting potential sex-specific dynamics.

Collectively, these findings provide strong support for the involvement of *GDAP1* and *HECW2* in the epigenetic landscape of AUD, highlighting both shared and sex-dependent roles.

### 3.2. Postmortem Brain Tissue and Directionality of DNAm Effects

In postmortem brain tissue, increased DNAm associated with AUD was initially observed for both genes but was not replicated in a larger cohort. When exploring whether these epigenetic changes translated to alterations in gene expression, we found reduced *HECW2* expression in the discovery cohort, but not in the replication cohort. Notably, Lohoff et al. (2021), who identified *HECW2* as one of their most robust findings in blood DNAm analyses, detected no DNAm differences in postmortem brain tissue, underscoring potential tissue-specific methylation patterns [18]. Querying a recent cross-species meta-analysis [29] similarly did not reveal significant expression changes for *GDAP1* or *HECW2*, further suggesting their role in AUD may be primarily epigenetic.

The lack of replicability may be attributed to differences in sample characteristics, such as brain pH and PMI, which, while not directly correlated with DNAm levels, could interact with other unmeasured factors. Another explanation lies in the dynamic nature of DNAm, which may vary based on chronicity, environmental influences, or comorbidities. While acute alcohol use may globally reduce DNAm, chronic consumption is often linked to hypermethylation in various brain regions [8]. This dual effect could contribute to inconsistencies across cohorts.

Interestingly, DNAm directionality differed between tissues, with hypomethylation in blood but hypermethylation in brain tissue. Previous rat studies have similarly shown tissue-specific responses to alcohol exposure, for example, with increased methylation in some brain regions but decreased levels in the liver [30,31]. These findings emphasize the need for multi-tissue studies to fully unravel the epigenetic mechanisms underlying AUD.

### 3.3. Insights from the Rat Model

To further explore the tissue-specific effects of AUD, we analyzed DNAm in a rat model, that enabled paired comparisons of blood and brain tissue, focusing on the prelimbic and infralimbic regions of the medial prefrontal cortex. The medial prefrontal cortex plays an important role in regulating approach and avoidance behavior, and epigenetic modifications in this region have been implicated in the development of AUD [32,33].

Unlike in humans, no differential DNAm was observed in rat blood. However, *HECW2* DNAm was significantly reduced in the prelimbic cortex, contrasting with previous reports of global DNA hypermethylation in this region in AUD [34], suggesting gene-specific regulation.

A recent study by Domi et al. (2024) demonstrated individual variability in addiction-related behavior among rats and linked neuroadaptations in the prelimbic cortex to the behavioral manifestation of AUD [35]. This interindividual variability may have obscured potential DNAm differences in *GDAP1*, warranting further investigation. Alternatively, *GDAP1* modifications may be human-specific, highlighting the need for cross-species studies to delineate conserved versus species-specific epigenetic mechanisms underlying AUD. These results underscore the tissue-specific nature of DNAm changes in the rat model, contrasting with the more systemic patterns observed in humans, where both blood and brain tissue showed alterations associated with AUD.

### 3.4. Limitations

Several limitations should be acknowledged when interpreting these findings. Small sample sizes in postmortem brain tissue may have reduced statistical power, affecting replicability. Additionally, postmortem samples often lack detailed social, environmental, and clinical information, hindering the consideration of factors known to influence DNAm. Incomplete data on potential confounders such as brain pH, PMI, blood alcohol levels, smoking, and RNA integrity further complicate interpretation, despite RNA integrity number (RIN)-adjusted analyses showing no significant differences.

The blood and postmortem brain cohorts originated from different populations (German and Australian, respectively), which may have introduced population-related variability. However, postmortem brain samples from the German cohort were not available, and the Australian samples provided the best-characterized resource for this analysis.

In the animal model, it should be noted that only male rats were used, which limits the generalizability of the findings and precludes assessment of potential sex-specific effects on DNAm. Moreover, gene expression analyses could also not be performed in the rat model, as RNA was not collected from the animal samples, limiting the ability to relate DNAm changes to transcriptional alterations.

In the human whole blood cohort, incomplete data on smoking, psychotropic medication, BMI, and alcohol consumption may introduce confounding effects. However, available data suggest no significant impact on *HECW2* or *GDAP1* DNAm. The high variability in *HECW2* DNAm across cohorts underscores the inherent challenges in interpreting epigenetic data, although outlier inclusion did not alter findings. The absence of RNA expression data in blood further limits functional insights.

A critical unresolved question is whether DNAm differences result from acute or chronic alcohol exposure, addiction processes, or predisposition contributing to disease susceptibility [36]. Addressing these limitations in future studies will be essential to fully elucidate the role of DNAm in AUD pathophysiology.

### 3.5. Conclusions and Outlook

This study provides robust validation of *HECW2* and *GDAP1* DNAm associations with AUD while revealing tissue-, species-, and sex-specific patterns. The opposing DNAm directionality in blood and brain highlights the complexity of alcohol-related epigenetic modifications. Future research should focus on larger, well-characterized cohorts, integrate multi-tissue and cross-species approaches, and include gene expression analyses to clarify the functional significance of DNAm changes. Longitudinal designs may help delineate whether these changes represent causes, consequences, or biomarkers of AUD. Future studies should also extend these analyses to female animals and include additional brain regions such as the amygdala, which may show even more pronounced sex-dependent epigenetic effects related to addiction processes. Elucidating the molecular mechanisms associated with these epigenetic modifications will be a key step toward understanding their role in AUD pathophysiology and identifying potential targets for therapeutic intervention.

## 4. Materials and Methods

### 4.1. Study Samples

#### 4.1.1. Human Whole Blood Samples

Samples were obtained from the Munich gene bank initiated in 1998 at the Psychiatric Department of the Ludwig-Maximilians-University [37,38]. The study sample comprised 258 patients (158 male, 100 female, mean age (±SD): 43.83 ± 10.98) being diagnosed with AUD according to the Diagnostic and Statistical Manual of Mental Disorders, Fourth Edition (DSM-IV) and 341 healthy control participants (159 male, 182 female, mean age (±SD): 42.57 ± 15.93). For the patients, the daily amount of alcohol consumed was assessed in grams for N = 249 patients, smoking behavior was assessed as the number of cigarettes consumed daily for N = 237 patients, BMI was available for N = 248 patients and information on the use of psychotropic medication was available for N = 239 patients. Of these, 133 patients (55.6%) were under current psychotropic medication. No information on these variables was available for control participants. All subjects gave written informed consent to participate in the study, which was approved by the ethics committee of the University of Munich.

#### 4.1.2. Human Postmortem Brain Samples

Human brain tissue samples (Brodmann area 9) were obtained from the New South Wales Brain Tissue Resource Centre at the University of Sydney, Australia (NSWBTRC) in two cohorts, namely a discovery cohort with tissue samples from 13 male participants with AUD and 10 male control participants and a larger replication cohort with samples from 55 participants with AUD (38 male, 17 female) and 64 control participants (47 male, 17 female).

Classification of AUD and control participants was done postmortem by next-of-kin interviews using the Diagnostic Instrument for Brain Studies-Revised, which is consistent with the criteria of the DSM-IV [39]. Participants with AUD had consumed 50 g or more ethanol on a daily basis [40], whereas control participants had consumed less than 20 g ethanol per day. All participants were of Caucasian descent. The sample demographics can be extracted from Supplementary Table S1. All experiments with human postmortem brain tissue were approved by the Institutional Review Board (2021-681-MA).

#### 4.1.3. Induction of Alcohol Dependence in Rats

Food and water were available ad libitum. Holding rooms for all animals were kept under controlled conditions of light (12 h light-dark cycles from 07:00 to 19:00), temperature (20–22 °C) and humidity (65%). Animals were sacrificed during the active cycle and experimental procedures approved by the local animal care committee (Regierungspräsidium Karlsruhe, Germany, 35-9185.81/G-166/21). Blood and brain samples were obtained from the chronic intermittent ethanol exposure (CIE) rat model that induces aspects of alcohol dependence in animals, described in more detail by Meinhardt & Sommer (2015) [41]. Briefly, to induce alcohol dependence, male Wistar rats (Charles River Laboratory) had been exposed to daily intermittent cycles of 16 h alcohol vapor exposure and withdrawal over 7 weeks. Control animals were housed in the same room, but were exposed to air only throughout the entire experiment. Rats were weight-matched and assigned to two groups which were either exposed to ethanol vapor (n = 15) or normal air (n = 16). From each animal, blood samples as well as brain samples from the infralimbic and prelimbic cortex were obtained one day after alcohol abstinence. Brain tissue was flash frozen in isopentane and stored at −80 °C until further processing. Brains were dissected using a cryostate and the brain regions of interest, infralimbic and prelimbic cortex, were extracted out of 100 µm brain slices using a micropuncher.

### 4.2. DNAm Analysis

#### 4.2.1. Human Whole Blood Samples

Genomic DNA was isolated from whole blood by standard procedures. 500 ng genomic DNA was bisulfite converted using the EpiTect Fast Bisulfite Kit (Qiagen, Hilden, Germany) according to the manufacturer’s instructions. Bisulfite converted DNA was eluted in 20 µL elution buffer and stored at −20 °C until further analysis. Region-specific PCRs were conducted using the PyroMark PCR Kit (Qiagen, Hilden, Germany) according to manufacturer’s instructions to amplify an intragenic region of *HECW2* spanning cg18752527 (for primer sequences see [14]) and a region within the *GDAP1* gene promoter spanning amongst other CpG sites cg23779890 (hg19 reference genome coordinates: chr22:19,950,054-19,950,064; PCR forward primer: 5-ATTTTTAGGTTTGTTAGGGGTTTTTTAGT-3, PCR reverse primer: 5-Biotin-ACTTCTCCCTCCCACACTACCC-3) [22,23]. Successful amplification and specificity of the PCR products was verified and visualized via agarose gel electrophoresis. DNA methylation (DNAm) was analyzed by pyrosequencing (for *HECW2* sequencing primer see [14], *GDAP1* sequencing primer: 5-GTTTGTTAGGGGTTTTTTA-3) using the PyroMark Q24 system and the corresponding PyroMark Q24 Software 2.0 (Qiagen, Hilden, Germany). Each sample was amplified twice and both amplicons sequenced as technical replicates. The mean percentage was used for further analysis. However, replicates revealing a deviation ≥ 3% were repeated. To detect disparate amplification of unmethylated DNA fragments a titration assay using standardized bisulfite-converted control DNA samples (EpiTect Control DNA, Qiagen, Hilden, Germany) with established DNAm levels of 0%, 25%, 50%, 75% and 100% DNAm was performed. For *GDAP1*, DNAm of the three CpG sites analyzed were highly correlated (r > 0.88, *p* < 0.001). Hence, all statistical analyses were performed using the mean DNAm.

#### 4.2.2. Human Postmortem Brain Samples

DNA and RNA from BA9 were extracted according to [16,17]. Genomic DNA was isolated using the QIAamp DNA micro kit (Qiagen, Hilden, Germany) according to the manufacturer’s protocol. Further sample processing to obtain DNAm levels in *GDAP1* promoter region and *HECW2* intragenic region was performed identical to human whole blood analyses.

#### 4.2.3. Rat Blood and Brain Samples

Genomic DNA was isolated using the DNeasy extraction kit (Qiagen, Hilden, Germany) according to standard procedures. Equivalently to human whole blood analyses, samples were bisulfite converted and region-specific amplifications were performed. However, primer sequences were adapted to the rat genome. The *Gdap1* promoter sequence showed no homology in the investigated region. Therefore, a CpG site in similar distance to the transcription start site was examined (PCR forward primer: 5′-GGGTGTTTTTATGTTTAAGTAAAGTTTAAAG-3′, PCR reverse primer: 5′-Bio-AAAATATAATTTCACCCCAAAAAAACTAT-3′). Although there exists a homologous region in rat DNA sequence to the investigated intragenic region in *HECW2* in humans, the CpG site of interest is not present in rat DNA. Therefore, a CpG site 15 bp upstream, which is present in rat but not human DNA sequence was investigated (PCR forward primer: 5′-GGGAATATTTTTTATAATGTAGTTTTAATTGTG-3′, PCR reverse primer: 5′-Bio-CACTCCATATTTTCATTTACTTATCATAACAAC-3′). As DNA yields were low, nested PCRs were performed using the same primer pairs. Pyrosequencing was performed as described for the human samples, except for the sequencing primers used (*Gdap1* sequencing primer: 5′-GAAAAATTTTTTATTTTATTATATT-3′, *Hecw2* sequencing primer: 5′-AAATAGAATGTTTTTTTAGATAT-3′).

### 4.3. Gene Expression Analysis

Gene expression of *GDAP1* and *HECW2* in human postmortem brain tissue was assessed using quantitative real-time PCR (qRT-PCR) with two primer pairs for each gene as described previously [42,43,44]. RNA was extracted using Trizol and the RNAeasy Mini kit (Qiagen, Hilden, Germany) following manufacturer’s instructions. Quality check was performed by measuring 260/280 ratios on a Nanodrop (Thermo Fisher Scientific, Waltham, MA, USA) and RNA integrity using the Agilent 2100 Bioanalyzer (Agilent, Santa Clara, CA, USA) for N = 113 samples. The mean RIN (±standard deviation) for these samples was 8.11 (±1.00). 100 ng RNA were then reverse transcribed into cDNA using reverse transcription kits according to manufacturer’s instructions (Thermo Fisher Scientific, Waltham, MA, USA). For qRT-PCR, Power SYBR Green PCR Master Mix (Thermo Fisher Scientific, Waltham, MA, USA) was used in a total reaction volume of 20 µL. Measurements were taken on an Applied Biosystems 7900 HT RT-PCR System with 40 cycles of 95 °C for 15 s followed by 60 °C for 1 min. As a quality control measure, melting profiles were recorded at the end of each run to assure aberrant fragment amplification. Primers were designed with one pair targeting closer to the mid of the gene sequence and one toward the 3′ end (sequences are listed in Supplementary Table S2) with an amplicon length of 291–376 bp. Expression levels were normalized to the housekeeping genes *GAPDH*, and *ALUSX*. All genes were run in technical duplicates. Gene expression levels were normalized to the control participants. There were four technical drop-outs for *GDAP1* for the first primer pair.

Further information is provided in the Supplementary Materials with the results of the additional primer pair and housekeeping gene provided in Supplementary Table S3 and their correlations in Supplementary Figures S2 and S3. Supplementary Table S4 shows the correlation of the RIN with gene expression.

### 4.4. Statistical Analysis

Statistical analyses and data visualization were conducted using R (version 4.2.2) [45]. Linear models were fitted with generalized least squares (GLS) using the nlme package [46] to evaluate the effects of AUD and, where applicable, sex on DNAm in human blood and postmortem brain tissue, as well as gene expression in human brain tissue. GLS models were also used to assess DNAm differences across rat blood and brain tissue with AUD as the factor, including age as a covariate when applicable. Unequal variances were addressed by incorporating a weighting term to model within-group heteroscedasticity when relevant.

Outliers, defined as values exceeding 3 standard deviations from the mean, led to the exclusion of 3 participants (1 male, 2 female) for *GDAP1* and 10 participants (3 male, 7 female) for *HECW2* DNAm analyses in the human blood cohort. Results without outlier removal are in the Supplementary Materials and Supplementary Figure S1.

Post hoc *t*-tests were used to further investigate interaction effects. For the human whole blood cohort, the daily amount of alcohol consumed, BMI, smoking behavior and psychotropic medication were only available in participants with AUD, which is why post hoc *t*-tests (psychotropic medication) and Pearson’s correlation (alcohol consumed, BMI and smoking) were used to assess any association of these factors with DNAm. Welch’s *t*-test was applied when variances were unequal.

Statistical significance was defined as *p* < 0.05. Where applicable, *p*-values were adjusted for multiple comparisons using the Bonferroni correction.

## Data Availability

The original contributions presented in this study are included in the article and Supplementary Materials. Further inquiries can be directed to the corresponding author.

## Supplementary Materials

The following supporting information can be downloaded at: https://www.mdpi.com/article/10.3390/ijms262210840/s1.

## Abbreviations

The following abbreviations are used in this manuscript:

AUD	Alcohol use disorder
DNAm	DNA methylation
DALYs	Disability-adjusted life years
EWAS	epigenome-wide association study
GDAP1	Ganglioside-induced Differentiation Associated Protein 1
HECW2	HECT, C2 and WW domain containing E3 ubiquitin protein ligase 2
SD	Standard deviation
BMI	Body mass index
Ctrl	Controls
PMI	Postmortem interval
DSM-IV	Diagnostic and Statistical Manual of Mental Disorders, Fourth Edition
CIE	Chronic intermittent ethanol exposure
qRT-PCR	Quantitative real-time PCR
RIN	RNA integrity number
GLS	Generalized least squares
