# Supplementary material for: Validation of GDAP1 and HECW2 as Epigenetic Markers of Alcohol Use Disorder in Blood and Brain
Source: Int J Mol Sci. 2025 Nov 8;26(22):10840. doi: 10.3390/ijms262210840 (PMC12652996; doi:10.3390/ijms262210840)
Supplement: Supplementary file 1 [file ijms-26-10840-s001.zip › ijms-3941412-supplementary.pdf]

Validation of *GDAP1* and *HECW2* as epigenetic markers of alcohol use disorder in blood and brain (Wiegand et al.)

Supplementary Information

Supplementary Methods

Study Samples

Supplementary Table S1. Descriptive statistics of demographic data of the human postmortem brain sample.

	**Discovery**		**Statistics**	**Replication**		**Statistics**	**Statistics**
	Controls	AUD	Discovery	Controls	AUD	Replication	Disc. vs. Repl.
**N**	10	13		64	55		
**Age**	56.6 (±16.0)	51.4 (±13.2)	$t(21)=0.86$, $p=0.40$	56.1 (±10.9)	56.4 (±10.2)	$t(117)=-0.17$, $p=0.87$	$t(26.76)=-0.82$, $p=0.42$
**Sex (M/F)**	10/0	13/0	-	47/17	38/17	$\chi(1)^2=0.27$, $p=0.60$	-
**Brain pH**	6.6 (±0.3); N=10	6.3 (±0.4); N=12	$t(20)=1.43$, $p=0.17$	6.6 (±0.3); N=63	6.5 (±0.3); N=55	$t(116)=2.12$, $p=0.036$	$t(138)=-1.83$, $p=0.07$
**PMI (hours)**	32.8 (±12.2), N=10	34.8 (±19.1), N=13	$t(21)=-0.30$, $p=0.77$	30.1 (±15.8), N=63	34.0 (±14.6), N=55	$t(116)=-1.39$, $p=0.17$	$t(139)=0.57$, $p=0.57$
**Blood alcohol level (yes/no)**	0/3, N=3	6/6, N=12	$\chi(1)^2=2.50$, $p=0.11$	2/21, N=23	15/21, N=36	$\chi(1)^2=7.44$, $p=0.006$	$\chi^2(1)=0.70$, $p=0.40$
**Smoking (yes/no/ exsmoker)**	2/4/3; N=9	9/1/0, N=10	$\chi(2)^2=9.23$, $p=0.010$	20/27/10, N=57	33/11/2, N=46	$\chi(2)^2=14.45$, $p<0.001$	$\chi^2(2)=0.31$, $p=0.86$

Data are presented as count and/or mean (±SD). Counts were given when data were only available for a subsample. PMI: post-mortem interval, brain pH: pH-value of the brain, Disc.: discovery cohort, Repl.: replication cohort.

Supplementary Table S2. Human-specific forward and reverse primer sequences.

Name	Sequence forward	Sequence reverse	Product length
GDAP1 (primer pair 1)	5'-TTT ATG CGT TTG AAC TCA ACT-3'	5'-AAT TTG GCT ACG AAT CCT-3'	291 bp
GDAP1 (primer pair 2)	5'-AAA CGG AAA GCG ACC AAA C-3'	5'-GGA TGA GCT GCC ACG ACA AGA-3'	303 bp
HECW2 (primer pair 1)	5'-GGG CAG GAG AGA CGG TC-3'	5'-CCC TGG CAT GTC CTC GTC ATC-3'	295 bp
HECW2 (primer pair 2)	5'-CCC AAA GTA TTA AAG CTA ACC-3'	5'-CGA AAG CCA AAT AAA CGA CTC-3'	376 bp
GAPDH	5'-CAT GAG AAG TAT GAC AAC AGC CT-3'	5'-AGT CCT TCC ACG ATA CCA AAG T-3'	113 bp
ALUSX	5'-GAG GCT GAG GCA GGA GAA TCG-3'	5'-GTC GCC CAG GCT GGA GTG-3'	87 bp

Supplementary Results

DNAm Levels in Human Whole Blood – Including Outliers

Alcohol use disorder (AUD) patients showed reduced *GDAP1* DNAm compared to control participants (F(1,594)=9.92, p=0.002, η^2=0.02). There was no significant main effect of sex ((F(1,594)=3.42, p=0.07), but an interaction between AUD and sex (F(1,594)=4.02, p=0.046, η^2=0.01). Post-hoc t-tests showed that reduced DNAm was only present in male (t(315)=3.69, p<0.001, Cohen's d=0.41, mean±SD $DNAm_{Ctrl,male}$=5.53%±1.50%, mean±SD $DNAm_{AUD,male}$=4.93%±1.40%) but not in female participants (t(280)=0.37, p=0.71, mean±SD $DNAm_{Ctrl,female}$=5.55%±1.71%, mean±SD $DNAm_{AUD,female}$=5.47%±1.52%) (Supplementary Fig. S1A). Moreover, there was a significant association of *GDAP1* DNAm with age (F(1,594)=5.98, p=0.015, η^2=0.01), driven by higher *GDAP1* DNAm in older participants (r=0.09, p=0.024).

For *HECW2*, there was a significant main effect of AUD on DNAm (F(1,594)=14.18, p<0.001, η^2=0.02) and a significant main effect of sex (F(1,594)=5.80, p=0.016, η^2=0.01). There was no interaction between these two factors (F(1,594)=1.33, p=0.25). *HECW2* DNAm was decreased in both male (t(295.9)=2.79, p=0.006, Cohen's d=0.31, mean±SD $DNAm_{AUD,male}$=7.88%±5.48%) and female (t(279.4)=3.38, p<0.001, Cohen's d=0.36, mean±SD $DNAm_{AUD,female}$=8.51%±4.83%) AUD patients compared to control participants (mean±SD $DNAm_{Ctrl,male}$=9.41%±4.26%, mean±SD $DNAm_{Ctrl,female}$=11.17%±8.40%) (Supplementary Fig. S1B).

There were no linear effects of patients' daily drinking quantity on DNAm, neither for *GDAP1* (*r*=-0.06, *p*=0.37) nor for *HECW2* DNAm (*r*=-0.03, *p*=0.67). Moreover, there was no difference with respect to psychotropic medication in patients, neither in *GDAP1* (*t*(236)=-0.60, *p*=0.55) nor in *HECW2* DNAm (*t*(236)=-0.32, *p*=0.75). There was also no correlation of DNAm with the number of cigarettes consumed daily in patients, neither in *GDAP1* (r=-0.08, *p*=0.23) nor in *HECW2* (r=-0.05, *p*=0.40). Lastly, there was no correlation between DNAm and BMI in patients, neither in *GDAP1* (r=0.08, *p*=0.19) nor in *HECW2* (r=-0.05, *p*=0.40).

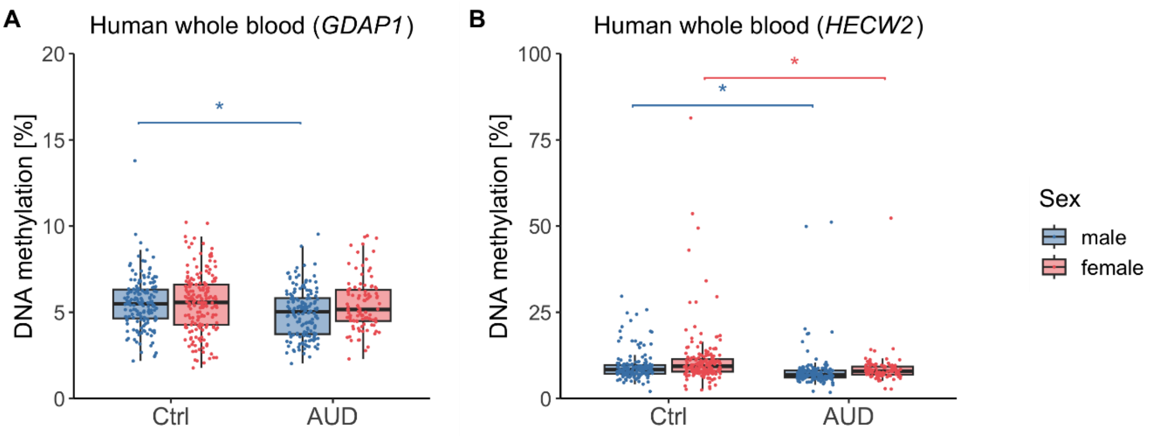

Supplementary Fig. S1. Human whole blood DNA methylation in AUD patients and control participants. DNAm distributions in male and female participants within the *GDAP1* gene promoter region (A) and *HECW2* intragenic region (B).

DNAm Levels in Human Postmortem Brain Samples - Associations with Confounding Factors

Investigating the effect of further potentially confounding factors on DNAm revealed no significant associations with PMI (*GDAP1*: r=0.39, *p*=0.06; *HECW2*: r=-0.07, *p*=0.74), brain pH (*GDAP1*: r=0.03, *p*=0.91; *HECW2*: r=-0.11, *p*=0.64), blood alcohol at death (*GDAP1*: *F*(1,13)=4.48, *p*=0.05; *HECW2*: *F*(1,13)=0.02, *p*=0.90) and smoking (*GDAP1*: *F*(2,16)=1.13, *p*=0.35; *HECW2*: *F*(2,16)=3.14, *p*=0.07) in the discovery cohort.

Similarly, in the replication cohort no significant associations of DNAm with PMI (*GDAP1*: r=-0.13, *p*=0.17; *HECW2*: r=-0.004, *p*=0.97), blood alcohol at death (*GDAP1*: *F*(1,57)=0.18, *p*=0.67; *HECW2*: *F*(1,57)=0.51, *p*=0.48) and smoking (*GDAP1*: *F*(2,99)=0.65, *p*=0.52; *HECW2*: *F*(2,99)=0.08, *p*=0.92) were found. While there was also no correlation of brain pH with *GDAP1* DNAm (r=-0.12, *p*=0.18), there was a nominal significant correlation of *HECW2* DNAm and brain pH (r=-0.19, *p*=0.038), which, however would not survive multiple testing.

Gene Expression in Human Postmortem Brain Samples

To increase reliability, gene expression analyses were performed using two different primer pairs and two different house-keeping genes, namely *glycerinaldehyde-3-phosphate-dehydrogenase* (*GAPDH*) and *ALUSX*. Supplementary Table S2 shows the results for the second primer pair and the house-keeping gene *ALUSX* and Supplementary Figures S2 and S3 depict the correlation between the data using different primer pairs and housekeeping genes.

Supplementary Table S3. Results of the gene expression analysis using an alternative primer pair and different housekeeping genes, namely *GAPDH* and *ALUSX*.

		**Discovery cohort**			**Replication cohort**		
		Primer pair 1 (*ALUSX*)	Primer pair 2 (*GAPDH*)	Primer pair 2 (*ALUSX*)	Primer pair 1 (*ALUSX*)	Primer pair 2 (*GAPDH*)	Primer pair 2 (*ALUSX*)
GDAP1	AUD	$F(1,17)=0.85$, $p=0.37$	$F(1,20)=0.82$, $p=0.38$	$F(1,20)=1.26$, $p=0.28$	$F(1,113)=1.86$, $p=0.18$	$F(1,114)=0.38$, $p=0.54$	$F(1,114)=0.69$, $p=0.41$
	Sex	-	-	-	$F(1,113)=4.49$, $p=0.036$	$F(1,114)=1.31$, $p=0.25$	$F(1,114)=3.68$, $p=0.06$
	AUD*Sex	-	-	-	$F(1,113)=0.09$, $p=0.76$	$F(1,114)=0.14$, $p=0.71$	$F(1,114)=0.02$, $p=0.89$
	Age	$F(1,17)=3.07$, $p=0.10$	$F(1,20)=2.98$, $p=0.10$	$F(1,20)=7.34$, $p=0.014$	$F(1,113)=5.03$, $p=0.027$	$F(1,114)=2.16$, $p=0.14$	$F(1,114)=11.48$, $p=0.001$
HECW2	AUD	$F(1,20)=4.16$, $p=0.055$	$F(1,20)=0.81$, $p=0.38$	$F(1,20)=1.30$, $p=0.27$	$F(1,114)=6.18$, $p=0.014$	$F(1,114)=0.98$, $p=0.32$	$F(1,114)=1.47$, $p=0.23$
	Sex	-	-	-	$F(1,114)=3.70$, $p=0.06$	$F(1,114)=0.11$, $p=0.74$	$F(1,114)=1.50$, $p=0.22$
	AUD*Sex	-	-	-	$F(1,114)=0.10$, $p=0.74$	$F(1,114)=0.63$, $p=0.43$	$F(1,114)=0.57$, $p=0.45$
	Age	$F(1,20)=8.10$, $p=0.010$	$F(1,20)=3.85$, $p=0.06$	$F(1,20)=8.47$, $p=0.009$	$F(1,114)=5.51$, $p=0.021$	$F(1,114)=0.63$, $p=0.43$	$F(1,114)=0.15$, $p=0.70$

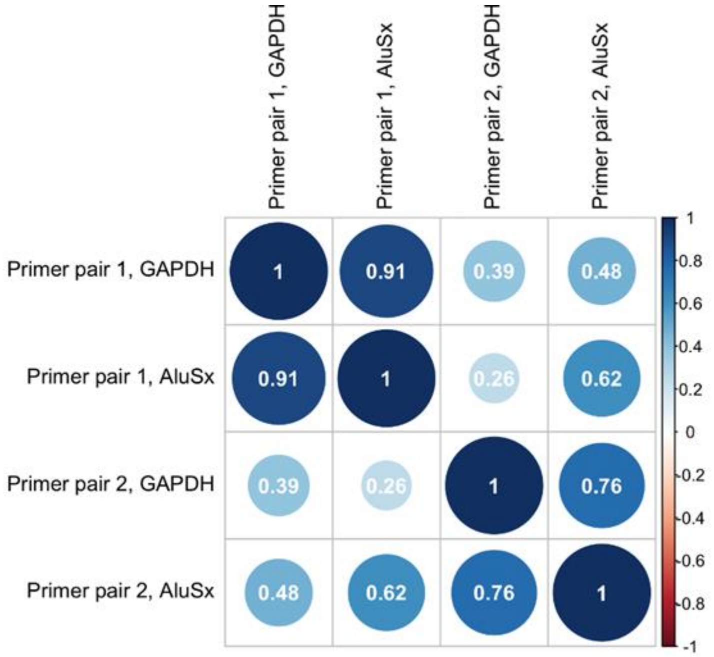

Supplementary Fig. S2. Correlation of gene expression data derived from different primer pairs and housekeeping genes for *GDAP1*. Gene expression data was replicated by using two different primer pairs and two different housekeeping genes, namely *GAPDH* and *ALUSX*.

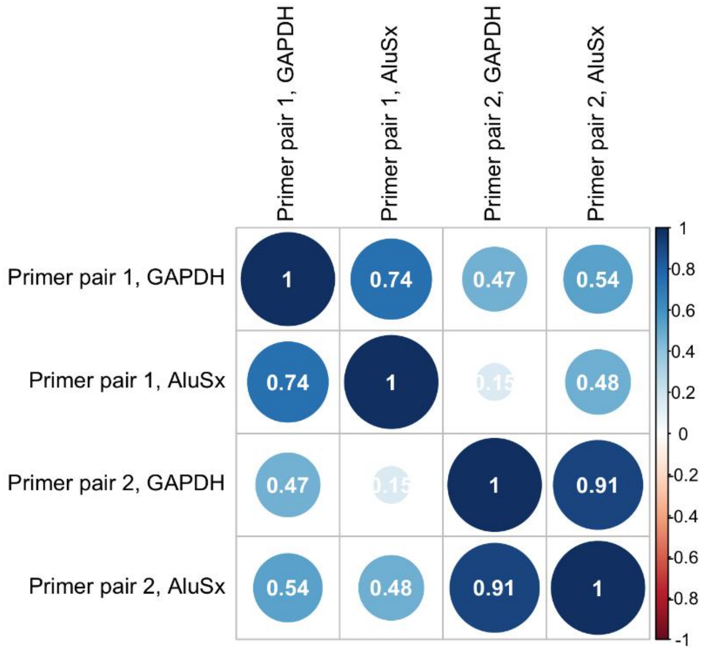

Supplementary Fig. S3. Correlation of gene expression data derived from different primer pairs and housekeeping genes for *HECW2*. Gene expression data was replicated by using two different primer pairs and two different housekeeping genes, namely *GAPDH* and *ALUSX*.

RNA Integrity Number (RIN)

Supplementary Table S3 shows the correlation of the RNA integrity number (RIN) with the gene expression levels.

Supplementary Table S4. Correlation of the gene expression with the RNA integrity number (RIN)

	**Primer pair 1 (*GAPDH*)**	**Primer pair 1 (*ALUSX*)**	**Primer pair 2 (*GAPDH*)**	**Primer pair 2 (*ALUSX*)**
GDAP1	r=-0.17, *p*=0.10	r=-0.30, *p*=0.003	r=-0.24, *p*=0.02	r=-0.47, *p*<0.001
HECW2	r=-0.45, *p*<0.001	r=-0.61, *p*<0.001.	r=-0.15, *p*=0.14	r=-0.34, *p*<0.001

Gene Expression in Human Postmortem Brain Samples – Analyses including RIN

The following results are based on a linear model with the factors *AUD* and, if applicable, *sex* and the covariates *age* and *RIN*. Hence, for these analyses only a subsample (N=113) is used for which the RIN is available. In the discovery cohort (N_{AUD} = 5, N_{Ctrl} = 5), gene transcription was reduced in participants with AUD for *HECW2* (*F*(1,6)=17.33, *p*=0.006, η^2=0.74) and but not for *GDAP1* (*F*(1,5)=1.14, *p*=0.33). There was no significant association between gene expression and age, neither for *HECW2* (*F*(1,6)=2.92, *p*=0.14) nor for *GDAP1* (*F*(1,5)=0.07, *p*=0.80) and no association between RIN and gene expression, neither for *HECW2* (*F*(1,6)=4.28, *p*=0.08) nor for *GDAP1* (*F*(1,5)=0.21, *p*=0.66).

In the replication cohort, there was no effect of AUD on gene expression, neither for *GDAP1* (*F*(1,85)=2.52, *p*=0.12) nor for *HECW2* (*F*(1,86)=0.73, *p*=0.39). Moreover, there were neither significant effects of age (*GDAP1*: *F*(1,85)=2.07, *p*=0.15; *HECW2*: *F*(1,86)=0.01, *p*=0.93) nor interactions of sex and AUD (*GDAP1*: *F*(1,113)=0.03, *p*=0.87; *HECW2*: *F*(1,86)=1.02, *p*=0.32). Moreover, for *GDAP1* there were no significant effects of sex (*GDAP1*: *F*(1,85)=1.56, *p*=0.22) and RIN (*GDAP1*: *F*(1,85)=0.89, *p*=0.35). However, for *HECW2* there were significant effects of both, sex (*F*(1,86)=4.07, *p*=0.047) and RIN (*F*(1,86)=21.74, *p*<0.001).

Gene Expression in Human Postmortem Brain Samples - Associations with Confounding Factors

Investigating the effect of further potentially confounding factors on gene expression revealed no significant associations with blood alcohol at death (*GDAP1*: *F*(1,9)=1.96, *p*=0.20; *HECW2*: *F*(1,10)=0.79, *p*=0.40) and smoking (*GDAP1*: *F*(2,14)=0.66, *p*=0.53; *HECW2*: *F*(2,16)=2.74, *p*=0.09) in the discovery

cohort. While there was no significant association of *HECW2* expression with PMI (r=-0.17, p=0.44), there was a significant association of *GDAP1* expression with PMI (r=-0.47 p=0.036). For brain pH, there was a significant association with *HECW2* expression (r=-0.68, p<0.001) but not with *GDAP1* expression (r=0.39, p=0.09).

Similarly, in the replication cohort no significant associations of gene expression with PMI (*GDAP1*: r=-0.12, p=0.19; *HECW2*: r=0.08, p=0.36), blood alcohol at death (*GDAP1*: $F(2,64)$=2.22, p=0.12; *HECW2*: $F(2,64)$=0.12, p=0.89) and smoking (*GDAP1*: $F(2,98)$=0.29, p=0.75; *HECW2*: $F(2,99)$=0.78, p=0.46) were found. However, there was a significant correlation of brain pH with gene expression for both genes (*GDAP1*: r=-0.33, p<0.001; *HECW2*: r=-0.47, p<0.001).
